# Relationship between Dietary and Other Lifestyle Habits and Cardiometabolic Risk Factors in Men

**DOI:** 10.1186/1758-5996-3-30

**Published:** 2011-11-14

**Authors:** Sayuri Katano, Yasuyuki Nakamura, Nagako Okuda, Yoshitaka Murakami, Nagako Chiba, Katsushi Yoshita, Taichiro Tanaka, Junko Tamaki, Toru Takebayashi, Akira Okayama, Katsuyuki Miura, Tomonori Okamura, Hirotsugu Ueshima

**Affiliations:** 1 Cardiovascular Epidemiology, Kyoto Women's University, Kyoto, Japan; 2 Department of Health Science, Shiga University of Medical Science, Otsu, Japan; 3 The First Institute of Health Service, Japan Anti-Tuberculosis Association, Tokyo, Japan; 4 Department of Medical Statistics, Shiga University of Medical Science Otsu, Japan; 5 Department of Health and Nutrition, Tsukuba International Junior College, Tsuchiura, Japan; 6 Department of Food Science and Nutrition, Graduate School of Human Life Science, Osaka City University, Osaka, Japan; 7 Department of Health Sciences, Interdisciplinary Graduate School of Medicine and Engineering, University of Yamanashi, Chuo, Japan; 8 Department of Public Health, Kinki University School of Medicine, Osaka-sayama, Japan; 9 Department of Preventive Medicine and Public Health, School of Medicine, Keio University, Tokyo, Japan

**Keywords:** alcohol, cardiometabolic risk factors, dietary intake, metabolic syndrome

## Abstract

**Background:**

Prevalence of men with cardiometabolic risk factors (CMRF) is increasing in Japan. Few studies have comprehensively examined the relation between lifestyles and CMRF.

**Methods:**

We examined the baseline data from 3,498 male workers ages 19 to 69 years who participated in the high-risk and population strategy for occupational health promotion (HIPOP-OHP) study at 12 large-scale companies throughout Japan. The physical activity of each participant was classified according to the International Physical Activity Questionnaire (IPAQ). Dietary intake was surveyed by a semi-quantitative Food Frequency Questionnaire. We defined four CMRF in this study as follows: 1) high blood pressure (BP): systolic BP ≥ 130 mmHg, or diastolic BP ≥ 85 mmHg, or the use of antihypertensive drugs; 2) dyslipidemia: high-density lipoprotein-cholesterol concentration < 40 mg/dl, or triglycerides concentration ≥ 150 mg/dl, or on medication for dyslipidemia; 3) impaired glucose tolerance: fasting blood sugar concentration ≥110 mg/dl; 4) obese: a body mass index ≥ 25 kg/m^2^.

**Results:**

Those who had 0 to 4 CMRF accounted for 1,597 (45.7%), 1,032 (29.5%), 587 (16.8%), 236 (6.7%), and 44 (1.3%) participants, respectively, in the Poisson distribution. Poisson regression analysis revealed that independent factors that contributed to the number of CMRF were age (b = 0.020, P < 0.01), IPAQ (b = -0.091, P < 0.01), alcohol intake (ml/day) (b = 0.001, P = 0.03), percentage of protein intake (b = 0.059, P = 0.01), and total energy intake (kcal)(b = 0.0001, P < 0.01). Furthermore, alcohol intake and its frequency had differential effects.

**Conclusions:**

Alcohol intake, percent protein and total energy intake were positively associated, whereas drinking frequency and IPAQ were inversely associated, with the number of CMRF.

## Background

The prevalence of men with cardiometabolic risk factors (CMRF) is increasing in Japan. CMRF such as high blood pressure (BP), dyslipidemia, impaired glucose tolerance (IGT), and obesity tend to cluster together and it is closely related to insulin resistance [1-4]. The clustering of CMRF is known as metabolic syndrome (MetS), and is thought to be related to daily lifestyle habits, including nutrient intake, physical activity, smoking and alcohol consumption. As for the association between alcohol consumption and MetS, there have been several cross-sectional studies. Some reported that the relation is inversely linear [5,6], J-shaped [7], or positively linear [8], whereas another found no relation [9,10]. Because alcohol consumption is associated with changes in consumption of several food groups and nutrients [11,12], an analysis of the relation between alcohol intake, as well as nutritional intake and CMRF, is needed. However, there are few comprehensive studies examining the relation between lifestyle factors and CMRF.

The objective of the present study was to examine cross-sectionally the relation between lifestyle habits including physical activity, smoking, dietary intake, alcohol intake and its frequency, and the number of CMRF in Japanese workers, and to find ways of preventing development of CMRF.

## Methods

### Participants

We analyzed baseline data from the high-risk and population strategy for occupational health promotion (HIPOP-OHP) study [13-16]. In brief, HIPOP-OHP was an interventional survey to establish a methodology for reducing cardiovascular disease (CVD) risk factors in the workplace. This study population consisted of full-time workers at 12 large-scale companies (mostly, manufactures of electric appliances) throughout Japan. Each company had 500-1,000 employees. Researchers followed the data of CVD risk factors, lifestyle and consciousness about health based on nutrition, physical activity and smoking for four years [13-16]. This study was performed as part of the management of safety and health with the approval of the Safety Hygiene Committee at each company. Accordingly, all employees were enrolled in this study. However, participation was voluntary, and we explained there was no need for participants to answer the required questionnaire if they did not want to. Approval for the study was obtained from the Institutional Review Board of Shiga University of Medical Science for ethical issues (No. 10-16). During 1999-2000, baseline data were collected from 7,346 male and female workers aged from 19 to 69 years old.

There were 4,261 participants (3,498 men and 763 women) aged 19 to 69 years (mean ± SD: 41.2 ± 9.6 years) who underwent a physical examination, a lifestyle survey and blood chemical examination. We excluded female participants because only about 20% of them were classified as drinkers; the amount of alcohol intake and its frequency were the important variables to examine in this study.

### Data collection and standardization

Physical and laboratory data were standardized according to the manual of the HIPOP-OHP research group [13]. Briefly, after a 5-min of silent rest measured by a sandglass, blood pressure was measured twice for each participant using the same automatic sphygmomanometer (Nihon Colin, BP-103iII) at each company, and the mean value was recorded. To measure the lipid concentrations in each participant, the company established a contract with a clinical laboratory; the blood testing was standardized through the US Cholesterol Reference Method Laboratory Network (CRMLN) [17]. The body mass index (BMI) was calculated as weight (kg) divided by height squared (m^2^).

Participants were asked about the type of, and time spent on, physical activities in their spare time for recreation, exercise or sport in the previous month. The physical activity of each participant was converted into MET-minutes per week (= MET level × minutes of activity/day × days per week) according to IPAQ [18], and participants were classified into four classes of physical activity: class 1: sedentary (<600 MET-minutes per week), class 2: some activity (<1500 but ≥ 600 MET-minutes per week), class 3: moderate activity (<3000 but ≥ 1500 MET-minutes per week), or class 4: high (≥ 3000 MET-minutes per week). Participants were also asked about their type of work (mostly sitting, mostly standing, work including heavy physical activity for about one hour, work including heavy physical activity for about 2 hours, or other types).

Drinking habit for each subject was assessed by a questionnaire common to all companies [14]. The frequency of alcohol intake during a typical week and the total alcohol intake on each occasion were determined and used to calculate the alcohol intake per week. This value was then divided by 7 to obtain the average alcohol intake per day. Subjects were asked to estimate their alcohol intake based on gou, a traditional Japanese drinking unit corresponding to 23 g of ethanol. One *gou *is equivalent to 2 US and UK drink units, or 180 ml of sake, and the ethanol content is roughly equivalent to that of a bottle of beer (663 ml), two single shots of whiskey (70 ml), a half glass of shochu (110 ml), or 240 ml of wine. Drinkers were defined as those consuming more than 0.3 *gou *(0.6 drinks) per week (1 g of ethanol a day).

We used a semi-quantitative food frequency method. Participants were asked about their average intake of food and beverages during a period of one or two months prior to the interview using the questionnaire, which consisted of 52 questions. Nutrient intake was calculated using the INTERMAP food table [19]. The calories from alcohol were counted in the total energy intake. Although the original version of our semi-quantitative food frequency method with 94 closed-ended questions has been validated [20], the shorter version with 52 questions used in the present study, has not been validated. The method of shortening of the original version of our FFQ with 94 questions involved mainly condensing several questions into a fewer questions. For example, the original version asked "how often do you eat blue fish (, red fish, white fish) per week?" in three questions. The shorter version asked "how often do you eat blue fish, red fish, or white fish per week?" in one question. If a participant usually ate blue fish, red fish, and white fish once each per week, he/she answered such in the original version. For the shortened version, he/she was instructed to answer that he/she ate these kinds of fish 3 times per week. Similar condensations were taken place mainly in questions related to bread, noodles, and soybean products consumption. Thus, actually omission was not performed. Participants' energy intakes were between 500 and 5000 kcal/day, and none of them were excluded from the study.

We defined four CMRF in this study according to previous studies [4,21,22] as follows: 1) high BP: SBP ≥ 130 mmHg, or DBP ≥ 85 mmHg,or the use of an antihypertensive drug; 2) dyslipidemia: either casual serum high-density lipoprotein-cholesterol (HDL) concentration < 40 mg/dl, or serum triglycerides (TG) concentration ≥ 150 mg/dl, or on medication for dyslipidemia; 3) IGT: fasting blood glucose concentration ≥110 mg/dl, or if less than 8 hours after meals≥140 mg/dl),or on medication for diabetes mellitus; 4) and obese: BMI ≥ 25 kg/m^2^.

### Statistical analysis

The chi-square statistical test for nominal variables and one way analysis of variance for continuous variables were performed to assess whether there were significant differences among the groups stratified by the number of CMRF. To obtain trend P, the "contrast" option for analysis of variance was used for continuous variables, and Mantel-Haensel test for prevalence variables. Partial correlation coefficients among lifestyle factors (IPAQ classification, alcohol intake [ml/day] and its frequency [times/week], percentage energy intake of protein, fat, and carbohydrate [%kcal], and total energy intake [mega calorie]) adjusted for age were obtained. Associations between the number of CMRF and lifestyle habits were analyzed by Poisson regression models, including age, IPAQ classification, type of work (mostly sitting, mostly standing, work including heavy physical activity for about one hour, work including heavy physical activity for about 2 hours, or other types; mostly sitting served as a reference), alcohol intake (ml/day), smoking (non-, past, or current smoker; non-smoker served as reference), percentage energy intake of protein, fat, and carbohydrate (%kcal), and total energy intake (kcal), as independent variables. Also the association between drinking frequency and the number of CMRF was analyzed by similar Poisson regression models by quintile of alcohol intake among 2,029 male drinkers. All P values were two-sided, and P < 0.05 was considered significant. All analyses were performed using SAS version 9.2 for Windows (SAS Institute, Cary, NC).

## Results

Among the 3,498 male participants, those who had 0 to 4 CMRF accounted for 1,597 (45.7%), 1,032 (29.5%), 587 (16.8%), 236 (6.7%), and 46 (1.3%) men, respectively, in the Poisson distribution. The mean total intake energy was 2,118 ± 465 kcal, and the percentage intakes of protein, fat and carbohydrate were 13.8 ± 1.7, 24.8 ± 4.6 and 60.6 ± 5.6%, respectively. Participants engaged in the following types of work: mostly sitting, mostly standing, work including heavy physical activity for about one hour, work including heavy physical activity for about 2 hours, or other types accounted for 49.3%, 37.3%, 5.2%, 3.6% and 4.5%, respectively. Characteristics of participants by group according to the number of CMRF are shown in Table 1. The mean SBP, DBP, BMI, alcohol intake, percentage of high BP, dyslipidemia, IGT, obese status, and of protein intake were higher among the groups with a higher number of CMRF. Mean IPAQ classification, alcohol drinking frequency, total energy intake, fat and percent carbohydrate intake, and prevalence of current smokers, were not different among the groups.

**Table 1:** Characteristics of Participants by Group According to the Number of Cardiometabolic Risk Factors - HIPOP-OHP Study

CMRF No	0	1	2	3	4	Total	P diff	P trend
Person N (%)	1597 (45.7)	1032 (29.5)	587 (16.8)	236 (6.7)	46 (1.3)	3498	-	
Age (year)	39.1 ± 9.6	41.8 ± 9.5	43.8 ± 8.	43.4 ± 9.4	46.3 ± 7.3	41.1 ± 9.6	<0.01	<0.01
SBP (mmHg)	110.2 ± 9.9	121.5 ± 14.7	130.8 ± 15.8	140.7 ± 14.1	142.4 ± 15.1	119.5 ± 16.3	<0.01	<0.01
DBP (mmHg)	67.3 ± 7.6	75.0 ± 10.5	81.6 ± 11.1	87.9 ± 10.1	89.8 ± 8.2	73.7 ± 11.6	<0.01	<0.01
High BP (%)	0	31.8	60.3	94.1	100	27.2	<0.01	<0.01
Dyslipidemia (%)	0	41.6	63.5	89.0	100	30.2	<0.01	<0.01
IGT (%)	0	5.7	15.7	27.1	100	7.5	<0.01	<0.01
BMI (kg/m^2^)	21.5 ± 1.9	23.2 ± 2.5	25.6 ± 3.0	27.2 ± 2.9	27.7 ± 1.9	23.2 ± 3.0	<0.01	<0.01
Obese (%)	0	20.9	60.5	89.8	100	23.7	<0.01	<0.01
IPAQ	1.4 ± 0.7	1.3 ± 0.7	1.3 ± 0.6	1.3 ± 0.7	1.3 ± 0.5	1.3 ± 0.7	0.21	0.25
Current-Smoking (%)	53.8	56.8	56.4	54.7	52.2	55.1	0.58	0.44
Alcohol (ml/day)	20.9 ± 30.3	24.2 ± 33.4	27.9 ± 36.5	23.2 ± 30.6	36.3 ± 40.4	23.4 ± 32.6	<0.01	<0.01
Alcohol (times/week)	2.9 ± 2.9	3.0 ± 3.0	3.1 ± 3.0	2.9 ± 2.8	3.1 ± 2.8	3.0 ± 2.9	0.71	0.78
Total energy (kcal)	2143 ± 449	2067 ± 461	2110 ± 484	2196 ± 508	2120 ± 481	2118 ± 465	<0.01	0.56
Protein (%kcal)	13.6 ± 1.6	13.9 ± 1.8	13.9 ± 1.6	14.0 ± 1.7	14.6 ± 1.9	13.8 ± 1.7	<0.01	<0.01
Fat (%kcal)	25.2 ± 4.5	24.7 ± 4.7	24.3 ± 4.7	24.7 ± 4.7	24.9 ± 5.1	24.8 ± 4.6	<0.01	0.66
Carbohydrate (%kcal)	60.5 ± 5.4	60.6 ± 5.8	60.9 ± 5.6	60.5 ± 5.7	59.3 ± 6.4	60.6 ± 5.6	0.35	0.15

Characteristics of participants by group according to the number of cardiometabolic risk factors (CMRF) in 3,498 Japanese men in 1999-2000 are shown. The chi-square statistical test for nominal variables and one way analysis of variance for continuous variables were performed to assess whether there were significant differences among the groups stratified by the number of CMRF. To obtain trend P, the "contrast" option for analysis of variance was used for continuous variables, and Mantel-Haensel test for prevalence variables. We defined four CMRF in this study as follows: 1) high BP: SBP ≥ 130 mmHg, or DBP ≥ 85 mmHg,or the use of an antihypertensive drug; 2) dyslipidemia: HDL < 40 mg/dl, or TG ≥ 150 mg/dl, or on medication for dyslipidemia; 3)IGT: fasting blood sugar concentration ≥110 mg/dl, or if less than 8 hours after meals ≥140 mg/dl), or on medication for diabetes mellitus; 4) obesity: defined as BMI ≥ 25 kg/m^2^.CMRF = cardiometabolic risk factors, SBP = systolic blood pressure, DBP = diastolic blood pressure, IGT = impaired glucose tolerance, BMI = body mass index, IPAQ = International Physical Activity Questionnaire classification.

Results of partial correlation coefficients among lifestyle factors and CMRF adjusted for age are shown in Table 2. IPAQ was positively associated with percent protein and fat intakes, total energy intake; inversely associated with current smoking and percent carbohydrate intake. Amount of alcohol intake was positively associated with alcohol drinking frequency, percent protein and fat intakes, and current smoking; inversely with percent carbohydrate and total calorie intakes. Alcohol drinking frequency was positively associated with percent protein and fat intakes, and current smoking; inversely with percent carbohydrate and total calorie intakes. Percent protein intake was positively associated with percent fat intake; inversely with current smoking, percent carbohydrate and total energy intakes. Percent fat intake was positively associated with total calorie intake; inversely with smoking and percent carbohydrate intake. Percent carbohydrate intake was positively associated with smoking; inversely with total calorie intake.

**Table 2:** Partial Correlation Coefficients among Lifestyle Factors and Cardiometabolic Risk Factors Adjusted for Age -- HIPOP-OHP Study

	IPAQ	Alcohol intake (ml)	Alcohol intake (times/week)	Protein(% energy)	Total fat (% energy)	Carbohydrate (% energy)	Total energy intake (calories)
Alcohol intake (ml)	-0.003	1.000					
Alcohol intake (times/week)	-0.014	0.719**	1.000				
Protein (% energy)	0.095**	0.162**	0.140**	1.000			
Total fat (% energy)	0.049**	0.108**	0.127**	0.411**	1.000		
Carbohydrate (% energy)	-0.057**	-0.151**	-0.159**	-0.675**	-0.926**	1.000	
Total energy intake (calories)	0.059**	-0.064**	-0.063**	-0.042*	0.210**	-0.135**	1.000
Smoking	-0.088**	0.122**	0.076**	-0.088**	-0.071**	0.066**	-0.023

Partial correlation coefficients among the lifestyle factors and metabolic risk factors adjusted for age are shown. * P < 0.05, **P < 0.01.

IPAQ = International Physical Activity Questionnaire classification.

Results of Poisson regression analysis are shown in Table 3. Independent factors that contributed to the number of CMRF were age (regression coefficient: b = 0.020, P < 0.01), IPAQ (b = -0.091, P < 0.01), alcohol intake (ml/day) (b = 0.001, P = 0.03), percentage of protein intake (%kcal)(b = 0.059, P = 0.01), and total energy intake (kcal)(b = 0.0001, P < 0.01). The contribution of current smoking was not statistically significant (b = 0.089, P = 0.05).

**Table 3:** Independent Factors that Contributed to the Number of Cardiometabolic Risk Factors -- Results of Poisson Regression Analysis

Variable	Regression coefficient	P
Age (year)	0.020	<0.01
IPAQ	-0.091	<0.01
Current smoking	0.089	0.05
Alcohol intake (ml/day)	0.001	0.03
Protein (%kcal)	0.059	0.01
Fat (%kcal)	-0.008	0.62
Carbohydrate (%kcal)	0.007	0.67
Total energy intake (kcal)	0.0001	<0.01

Results of analysis by Poisson regression models on associations between the number of CMRF and lifestyle are shown. Covariates included are shown in this Table plus type of work with regard to occupational physical activity (mostly sitting, mostly standing, work including heavy physical activity for about one hour, work including heavy physical activity for about 2 hours, or other types; mostly sitting served as a reference). IPAQ = International Physical Activity Questionnaire classification

Results of Poisson regression analysis on the association of alcohol drinking frequency with the number of CMRF by quintile of alcohol intake among 2,029 male drinkers are shown in Table 4. Cut off alcohol intake levels among the 5 groups were 12.5, 24.3, 39.7, and 62.5 ml/day. There were about 400 men in each group. Except for the lowest alcohol intake quintile group, drinking frequency was significantly inversely associated with the number of CMRF independent of the other factors.

**Table 4:** Association of Alcohol Drinking Frequency with the Number of Cardiometabolic Risk Factors by Quintile of Alcohol Intake Among 2,029 Male Drinkers -- Results of Poisson Regression Analysis

Alcohol intake (range, ml/day)	Person N	Regression coefficient of drinking frequency	P
1.25-12.5	405	-0.007	0.87
12.8-24.3	406	-0.074	<0.01
24.5-39.7	408	-0.13	<0.01
39.9-62.5	393	-0.109	<0.01
62.7-250.0	393	-0.086	0.047

Results of analysis by Poisson regression models on association between alcohol drinking frequency and the number of CMRF by quintile of alcohol intake among 2,029 male drinkers are shown. Covariates included besides alcohol intake frequency were age, IPAQ classification, type of work (mostly sitting, mostly standing, work including heavy physical activity for about one hour, work including heavy physical activity for about 2 hours, or other types; mostly sitting served as a reference), smoking (non-, past, or current smoker; non-smoker served as a reference), percentage energy intake of protein, fat, and carbohydrate (%kcal), and total energy intake (kcal). IPAQ = International Physical Activity Questionnaire classification.

## Discussion

We found significant associations between percent protein intake, as well as total energy intake and the number of CMRF. The latter finding was expected, but the former finding was rather unexpected in the context of recent favorable results regarding low carbohydrate, high protein diets [23,24]. In this study, the average total energy intake was 2,118 kcal, and the percentages of protein, fat and carbohydrate of total energy intake were 13.8, 24.8 and 60.6%, respectively. These values are consistent with the results of the Japanese National Health and Nutrition Examination Survey [25], which reported that the average total energy intake of men with age **≥ **20 years was 2,173 ± 605 kcal. A study by Pan et al. in a population-based sample of 2,811 Chinese middle-aged and elderly, suggested habitual soy protein intake in men, but not in women, was associated with the risk of MetS [26]. Azadbakht et al. found that increased red meat consumption was cross-sectionally associated with greater risk of MetS and inflammation in 482 Tehrani females aged 40-60 years [27]. Thus, the results of some studies agreed with ours. Further research is therefore needed incorporating nutritional analyses.

We found a significant positive association between alcohol consumption and the number of CMRF, whereas there was a significant inverse association between frequency of alcohol intake and the number of CMRF except for the lowest alcohol intake quintile group. Thus, when the amount of alcohol intake was relatively moderate, a higher drinking frequency was associated with a lower number of CMRF. Several cross-sectional studies have reported an association between alcohol drinking and MetS prevalence. However, they showed inconsistent findings [5-10]. Dixon et al. showed that light to moderate alcohol consumption had a favorable effect on metabolic risk factors and insulin resistance in severely obese patients with BMI>35 kg/m^2 ^[6]. However, in the Atherosclerosis and Insulin Resistance study of 391 healthy 58-y-old men, no significant difference was found in alcohol consumption between the subjects with MetS and those without risk factors [10]. Yoon et al. who studied 7,962 Korean men and women and found that alcohol consumption had a significant inverse relation with the odds ratio for low HDL cholesterol in all alcohol groups; however, an increasing dose-response relation was found between alcohol consumption and the odds ratio for MetS [7]. Fan et al. studied patterns of alcohol consumption and MetS, and found that the usual daily quantity of alcohol intake was positively associated with MetS, and that the frequency of binge drinking was also positively associated with MetS [8]. Thus, our findings are basically consistent with those of Fan et al.

In the present study, we found a significant inverse association between physical activities assessed by IPAQ and the number of CMRF among mostly middle-aged male participants. There are not many studies on the association between physical activity and CMRF or MetS. Dalacrote et al. studied 362 community-dwelling elderly people in southern Brazil, and found no significant association between MetS and the IPAQ level of physical activity in men and women [28]. In a study by Ekelund et al. in a population-based sample of 3,193 European youths, lower physical activity was independently associated with MetS after adjustment for sex, age, and other covariates [29], Hahn et al. in a population-based study of 1,653 elderly participants also found that intense physical activities significantly reduced the odds of having MetS [30]. Thus, studies with a larger number of participants found similar results to ours.

The strengths of our study include being population-based, large-scale, and multi-site with highly standardized methods. Since the study included men of a broad range of ages, findings are likely to be generalizable to middle-aged Japanese men. The study was limited by its cross-sectional design. Second, we did not measure waist circumference (WC). Although the measurement of WC is widely advocated as a simple anthropometric marker of health risk, there remains no uniformly accepted protocol. Mason et al. showed that the measurement site had an influence on the apparent prevalence of abdominal obesity (>88/102 cm), ranging from 23 to 34% in men and 31 to 55% in women [31]. Panoulas et al. showed a significant inter-operator variability that could lead to disagreement among operators regarding the presence of central obesity in 9% of the patients [32]. We showed in a population based study that BMI and WC correlated very well in men and women, and that BMI could be used instead of WC in a study when the latter was not available [33]. Third, we did not have a validation study for our semi-quantitative food frequency method. Although the original version of our semi-quantitative food frequency method with 94 closed-ended questions has been validated [20], the shorter version with 52 questions used in the present study, has not been validated. The method of shortening of the original version questions involved mainly condensing several questions into a fewer questions. Thus, actually omission was not performed. As indicated above, our nutritional values were consistent with the results of the Japanese National Health and Nutrition Examination Survey [25]. We think our shorter version was able to capture almost all the components of the diet. Forth, we cannot say how many criteria a person needs to have in order to have cardiometabolic syndrome. For this purpose, we may better to have evidence from prospective studies. We cannot obtain such evidence, since this study is a cross-sectional study. However, previous studies have shown that separate components of cardiometabolic syndrome are associated with a higher risk of coronary heart disease and stroke. Thus, we think prevention from developing each component is more important than dichotomizing people into two groups with and without the syndrome and preventing development of the syndrome.

The messages we obtained from our present study are: in order to prevent from developing each component of CMRF, we recommend to participate in physical activities and reduce intake of total energy and not to take a large amount of alcohol at a time.

In conclusion, alcohol intake, percent protein and total energy intakes were positively associated whereas drinking frequency and IPAQ were inversely associated, with the number of CMRF.

## List of abbreviations

BMI: body mass index; BP: blood pressure; CMRF: cardiometablic risk factors; CVD: cardiovascular disease; DBP: diastolic blood pressure; HDL: high-density lipoprotein-cholesterol; HIPOP-OHP: the high-risk and population strategy for occupational health promotion; IGT; impaired glucose tolerance; IPAQ: the International Physical Activity Questionnaire; MetS: metabolic syndrome; SBP: systolic blood pressure; TG: triglycerides;

## Competing interests

The authors declare that they have no competing interests.

## Authors' contributions

KS participated in analyzing and interpreting the data, and drafting and preparing the manuscript, YN participated in designing and conducting the study, interpreting the data, and editing the draft. TT, and TO participated in managing the dataset and analyzing the data. YM participated in statistical consultation, while 0NO, NC, KY, JT and ToT participated in developing and conducting the nutritional survey. TO, and KM participated in the physical activity survey, and AO participated designing and conducting the study. HU was the principal investigator, designing and conducting the study and analyzing and interpreting the data.

All authors read and approved the final manuscript.
